# In Situ Monitoring Network for Deposition Morphology and Residual Stress Reconstruction

**DOI:** 10.3390/ma19091785

**Published:** 2026-04-28

**Authors:** Yi Lu, Hairan Huang, Xinyi Huang, Chen Wang, Wenbo Li, Bin Wu

**Affiliations:** College of Mechanical and Electrical Engineering, Nanjing Forestry University, Nanjing 210037, China18451009486@163.com (H.H.); wangchen@njfu.edu.cn (C.W.);

**Keywords:** laser metal deposition, residual stress, machine vision, DeepLabv3+, morphology reconstruction, thermo-mechanical simulation, high nitrogen steel

## Abstract

In laser metal deposition (LMD), complex thermo-mechanical coupling and irregular layer morphology significantly affect residual stress distribution. However, most simulations rely on idealized geometries, limiting prediction accuracy. This study proposes a data-driven framework integrating in situ vision-based morphology reconstruction with thermo-mechanical simulation for high nitrogen steel (HNS). An improved DeepLabv3+ network is developed to extract deposition layer contours under strong illumination and spatter interference, achieving a mean intersection over union (mIoU) of 97.32% and an overall accuracy of 99.42%. The reconstructed morphology is incorporated into a finite element model to enable dynamic heat source tracking and realistic geometric representation. The proposed method demonstrates high morphology reconstruction accuracy, with all measurement errors controlled within 0.91%. The simulated temperature field agrees well with experimental measurements. Furthermore, the predicted residual stress distribution is consistent with X-ray diffraction (XRD) results under different laser power conditions. The results indicate that local surface morphology significantly influences stress concentration, with protrusion regions exhibiting stress peaks up to 989 MPa, markedly higher than those in concave regions. This study improves the accuracy of residual stress prediction in LMD by incorporating real morphology data and provides insight into the relationship between morphological features and stress evolution in additively manufactured HNS components.

## 1. Introduction

Laser Metal Deposition (LMD), as a representative Directed Energy Deposition (DED) additive manufacturing technology, achieves track-by-track and layer-by-layer material accumulation by using a laser beam to create a molten pool on the substrate surface while simultaneously delivering powder [1]. However, due to the complex non-steady thermo-mechanical coupling inherent in the LMD process, unavoidable heat accumulation and stress evolution during fabrication often lead to severe geometric distortion and internal defects in components. These issues may ultimately result in structural failure and significantly limit the industrial application of this technology [2]. Although numerical simulation has become a crucial tool for predicting residual stress and assessing process feasibility, traditional residual stress characterization methods, despite their high precision, typically suffer from long detection cycles, high equipment costs, and expensive testing procedures. Consequently, they struggle to meet the real-time requirements of in situ quality control in industrial production [3]. Therefore, it is necessary to overcome the limitations of traditional offline modeling by introducing advanced in situ monitoring technologies. Machine vision-based monitoring can be used to capture and reconstruct the actual morphology of the deposition layer, thereby enabling the establishment of thermo-mechanical simulation models based on real geometric features. This provides a critical pathway for reducing simulation errors and improving the accuracy of residual stress prediction [4]. This need is also reflected in recent review studies, which have identified simulation-based prediction, experimental validation, and process monitoring as major directions in residual stress research in metal additive manufacturing [5].

In this context, in situ monitoring technologies based on machine vision have emerged as a promising solution. These techniques enable real-time observation of molten pool morphology and the evolution of the deposition layer geometry through non-contact imaging [6]. However, the imaging environment in LMD industrial settings is extremely harsh, posing significant challenges for high-precision feature extraction. Intense radiation from high-energy lasers, together with the self-emission of the high-temperature molten pool, leads to severe illumination fluctuations and dynamic background noise. Under such complex conditions, traditional image processing methods, such as gradient-based operators and threshold segmentation, are often inadequate. Fang et al. [7] pioneered the use of U-Net-based convolutional neural networks to capture in situ molten pool features during the SLM process, demonstrating the feasibility of end-to-end networks for extracting deposition contours under complex backgrounds. However, due to the down-sampling operations and limited multi-scale feature representation in conventional convolutional architectures, this approach often fails to recover fine edge details, especially in low-resolution images. As a result, the accuracy of subsequent morphology reconstruction of the deposition layer is compromised.

Lin et al. [8] proposed a super-resolution reconstruction method for deposition layers based on dual-path deep learning to address this limitation, significantly improving spatial resolution and edge clarity through parallel feature extraction and high-frequency detail fusion. Although this approach improves static detail recovery in single frames, the dynamic evolution of the molten pool during continuous monitoring is often accompanied by severe illumination fluctuations, leading to inconsistency in inter-frame predictions. Zhu et al. [9] developed a Transfer Learning Enhanced Physics-Informed Neural Network (TLE-PINN) for molten pool morphology prediction in laser melting. By incorporating heat conduction equations into the loss function, this model improves prediction accuracy under small-sample conditions. However, the model is primarily based on idealized flat-surface boundary conditions and does not consider the dynamic effects of stochastic surface undulations during actual deposition. Asadi et al. [10] proposed an in situ monitoring and geometric statistical analysis method for directed energy deposition using deep convolutional neural networks. This method enables real-time extraction of geometric features, such as molten pool area and width, through pixel-level segmentation, and establishes a predictive model for deposition bead geometry. However, it mainly focuses on static analysis of single frames and does not explore the thermo-mechanical coupling mechanism between complex morphological fluctuations and residual stress during multi-layer deposition. In response to this issue, Zhang et al. [11] introduced a temporal adaptive module into the U-Net architecture and proposed an improved molten pool detection network for L-DED. By utilizing spatio-temporal information from video sequences, this approach effectively suppresses transient background noise and improves detection stability. However, stochastic spatter during deposition remains a major source of interference, often generating false edges. Gadde et al. [12] addressed this issue by applying deep learning to analyze spatter behavior and molten pool dynamics, achieving multi-target identification in complex flow environments. A key challenge in future research is to eliminate spatter interference in real time while maintaining high spatio-temporal segmentation accuracy. Overall, existing machine vision-based monitoring methods mainly focus on qualitative assessment of process stability and often lack fine-grained, pixel-level segmentation capability. As a result, they are insufficient for accurately characterizing the continuous and subtle morphological variations in the deposition surface [13]. Moreover, few studies have integrated high-fidelity in situ morphology reconstruction with thermo-mechanical simulation to provide deterministic geometric boundary conditions for residual stress prediction in LMD. Therefore, a method capable of linking real deposition morphology with thermo-mechanical modeling is still needed to improve the accuracy and physical fidelity of residual stress prediction.

**Figure 1:**
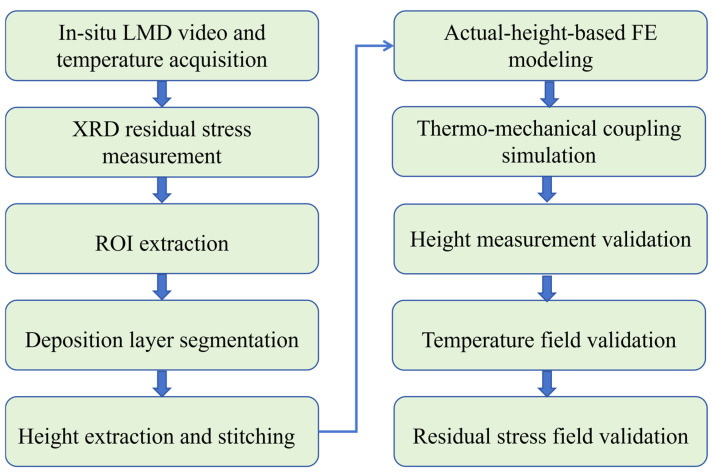
Overall methodological workflow of the proposed monitoring–reconstruction–simulation framework.

This study focuses on thin-walled circular HNS structures with significant surface undulations during LMD and proposes a residual stress prediction method that integrates machine vision monitoring with data-driven numerical simulation. First, an improved deep semantic segmentation network tailored to the intense illumination conditions of LMD was developed. By reconstructing the feature extraction backbone using a deep residual learning mechanism and designing a densely connected multi-scale feature fusion module, the network adopts a cascaded feature reuse strategy to enhance its ability to suppress high-intensity plume and spatter noise, thereby enabling pixel-level extraction of faint deposition edges. Second, based on the high-fidelity contour data acquired through vision, a thermo-mechanical coupled finite element model was established to reflect the actual surface waviness and overlapping features, thereby overcoming the accuracy limitations of traditional simplified models. On this basis, the evolution of temperature and stress fields during deposition under different laser powers was systematically investigated, with particular attention given to the mechanism by which surface geometric deviations induce localized residual stress concentration. Finally, the effectiveness and accuracy of the proposed method were validated by X-ray diffraction (XRD) measurements. The overall methodological workflow of the present study is illustrated in Figure 1.

## 2. Materials and Methods

### 2.1. Experimental Setup and Materials

To obtain the actual geometric morphology and transient thermal history during the deposition of thin-walled circular HNS structures, thereby providing accurate physical boundary conditions for subsequent thermo-mechanical coupled numerical simulations, an LMD experimental system integrated with visual monitoring and temperature measurement equipment was established. This platform was designed to capture real-time data on deposition layer morphology and thermal cycles. These data provide the experimental basis for geometric modeling and for validating the simulation results in the numerical analysis stage.

A specialized visual monitoring system was developed for in situ observation and data extraction of deposition surface morphology. The system comprises a CMOS industrial camera (Chameleon3 USB3, Teledyne FLIR, Wilsonville, OR, USA) and an 808 nm laser illumination system. It features a high image resolution (2048 × 1536 pixels) and a spatial resolution of 4.8 μm/pixel, enabling the capture of deposition dynamics at a frame rate of 30 fps. The camera exposure time was set to 10 ms to ensure sharp imaging during rapid dynamic processes. The optical path was integrated with a narrow-band pass filter (NBF), a neutral density (ND) filter, and K9 protective glass to attenuate interference from molten pool radiation and spatters, thereby ensuring the stability of the captured morphology and high image quality. Using this system, the evolution of the molten pool can be captured, and the geometric profile of the deposition layer can be monitored, providing high-precision geometric data for subsequent thermo-mechanical coupled simulations.

To synchronously record the transient thermal history during the deposition process, a two-color infrared pyrometer (IMPAC ISR 12-LO/GS, LumaSense, Raunheim, Germany) was employed. During the experiments, the pyrometer measurement spot was fixed on the substrate surface, with an effective spot radius of 1 mm.

**Figure 2:**
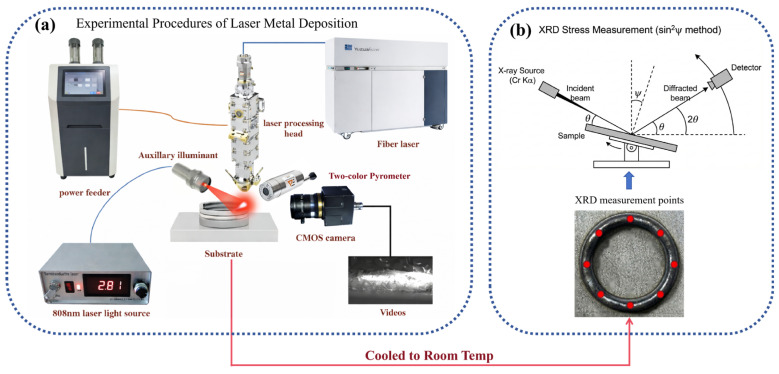
Experimental workflow: (**a**) in situ LMD monitoring system; (**b**) post-process XRD residual stress measurement and tangential sampling positions on the circular specimen after cooling to room temperature.

The experimental light source was a fiber laser (TruDiode 3006, TRUMPF, Ditzingen, Germany) with an operating wavelength of 1030 nm and a maximum output power of 3000 W. After collimation and focusing, the laser beam formed a spot with a diameter of 1 mm and irradiated the substrate surface through a coaxial powder feeding nozzle (YC52, Precitec, Gaggenau, Germany). The powder was delivered into the molten pool area by a powder feeder (RC-PGF-D, Raycham, Nanjing, China), with argon used as both the carrier gas and shielding gas, thereby ensuring a stable deposition process in an inert atmosphere. Sample preparation was carried out on a four-axis CNC stage (AFS-1200.80, Longyuan AFS, Beijing, China) integrated with an industrial turntable, enabling the deposition of circular tracks. After the samples cooled to room temperature, eight characteristic sampling sites were selected along the tangential direction of the circular ring. Residual stress at these locations was measured by XRD. The overall experimental workflow is illustrated in Figure 2.

The substrate material used in the experiments was 316 stainless steel with dimensions of 50 mm × 50 mm × 10 mm. The deposition material was HNS powder, and its chemical composition was adopted from Ref. [14], as listed in Table 1. The fabricated samples consisted of an eight-layer thin-walled circular structure with an outer diameter of 30 mm. Two laser power levels, 1000 W and 1500 W, were employed for sample preparation. Through systematic optimization, the process parameters were determined as follows: a scanning speed of 200 mm/min, a powder feed rate of 0.7 g/min, a carrier gas flow rate of 6 L/min, and a shielding gas flow rate of 10 L/min. These optimized parameters ensured uniform powder delivery while effectively preventing excessive melting and excessive thermal stress accumulation.

During the deposition process, a consistent scanning strategy was applied to each layer. Specifically, the laser head remained stationary while the industrial turntable rotated to form the circular track, during which the CMOS camera recorded in situ deposition images. Upon completion of each layer, the laser and powder feeder were simultaneously deactivated; however, the turntable continued rotating to capture the post-deposition morphology. After returning to the initial position, the system proceeded to deposit the subsequent layer. After the entire build was completed, the samples were allowed to cool to room temperature. Subsequently, X-ray diffraction was used to measure the tangential residual stress at selected points on the top surface, thereby ensuring the acquisition of high-fidelity stress data.

### 2.2. Deep Semantic Segmentation Model for Deposition Morphology Extraction

In LMD processes, the deposition surface is subject to intense illumination fluctuations and metallic reflection effects, which often result in insufficient contrast and overlapping grayscale distributions between the deposition region and the background, leading to blurred boundaries. Under such complex conditions, the backbone of the traditional DeepLabv3+ architecture has limited capability for capturing fine-grained structural features. In addition, its insufficient use of multi-scale information makes it difficult to achieve precise segmentation of the deposition region [15].

In view of this, the architecture of DeepLabv3+ was optimized in this study. First, to address the insufficient feature extraction capability of traditional shallow convolutional networks when processing industrial images with strong optical noise, a feature extraction backbone based on deep residual learning was constructed. The backbone adopts a super-deep residual architecture consisting of 101 convolutional layers to maximize network depth for capturing high-level semantic information without causing gradient vanishing. This architecture comprises four feature evolution stages and a total of 33 bottleneck residual units. By introducing identity-mapping shortcut connections, shallow spatial details are directly propagated to deeper layers, enabling the network to learn residual functions rather than the original mapping. Specifically, in the third feature evolution stage, the dense stacking of 23 residual units significantly expands the effective receptive field at this stage. The hundred-layer architecture further employs a “compression–convolution–expansion” bottleneck design to effectively reduce computational complexity and parameter redundancy. As a result, the network can robustly penetrate the high-intensity plume and spatter interference in the LMD process, extracting stable feature representations of deposition layer contours under complex illumination conditions, and significantly enhancing the model’s pixel-level perception of abrupt geometric variations [16].

Secondly, to overcome the limitations of fixed receptive fields when dealing with deposition layer morphologies at different scales, a Densely Connected Atrous Spatial Pyramid Pooling strategy was adopted in this study. Unlike traditional parallel and independent atrous convolutional branches, this module organizes atrous convolution layers in a cascaded manner. The output feature maps from a preceding layer with a smaller dilation rate are used as inputs for subsequent layers, while dense skip connections are established between different hierarchical levels. This design enables multi-level feature reuse along the channel dimension, effectively constructing a receptive field network with extremely high pixel sampling density. Through this dense connection mechanism, the network can adaptively aggregate multi-scale information ranging from local edge details to global contextual semantics. This effectively reduces the misclassification of spatter particles and ensures the continuity and integrity of the deposition layer contours under strong interference backgrounds [17].

**Figure 3:**
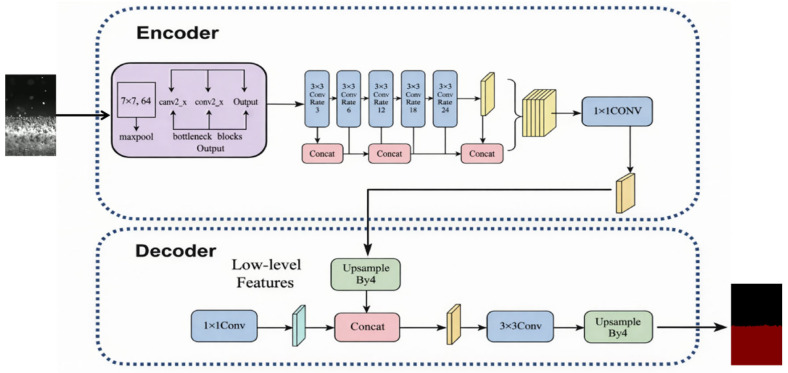
Architecture of the improved DeepLabv3+ network for deposition layer morphology extraction.

In addition, to further mitigate the loss of spatial information caused by repeated down-sampling operations, the decoder of the network was optimized by introducing a cross-scale semantic–spatial feature fusion mechanism. Instead of directly applying coarse up-sampling to high-level features, this mechanism employs lateral connections to fuse high-resolution spatial detail features preserved in the shallow layers of the backbone with the strong semantic features extracted from deeper layers through channel concatenation and convolution. This strategy effectively compensates for the deficiency of deep networks in edge localization, enabling the final segmentation masks to achieve both accurate semantic classification and pixel-level smooth geometric boundaries, while eliminating the jagged artifacts commonly observed in traditional methods [18]. Figure 3 illustrates the overall architecture of the improved network. Compared with the original DeepLabv3+, the improved model in this study includes three main modifications: (1) replacement of the backbone with a ResNet101-based deep residual architecture; (2) introduction of a densely connected atrous spatial pyramid pooling module; and (3) optimization of the decoder through cross-scale semantic-spatial feature fusion.

### 2.3. Deposition Contour Extraction and Morphology Reconstruction

During the LMD process, the solid–liquid interface is accompanied by intense overexposure, while the powder and spatter above introduce significant noise interference, making the extraction of the deposition layer surface contour highly challenging. To eliminate background noise interference and reduce the computational redundancy associated with full-image processing, a dynamic region of interest (ROI) extraction strategy was adopted in this study. Since the camera maintains a fixed relative position with respect to the laser head and moves upward synchronously, the relative position of the substrate in the image gradually shifts downward as the deposition layers accumulate.

To this end, during the processing of each layer, the lower boundary of the ROI was dynamically locked to the substrate position, while the upper boundary was adaptively adjusted to the top of the image. Under this strategy, although the width of the ROI remained constant, its height gradually increased with the number of deposited layers, thereby ensuring that the computational region continuously covered the effective material accumulation area. This dynamic adjustment mechanism effectively excluded irrelevant regions ahead of the molten pool and below the substrate, significantly reducing the instability in deposition layer height extraction and enabling the algorithm to focus on the actual morphological features.

After the ROI was determined, semantic segmentation was performed on the image using the improved DeepLabv3+ model. Sequential frames were extracted from deposition experiment videos of multiple specimens to train the model, and an initial dataset containing 1130 original images was constructed. Data augmentation techniques, including random rotation, flipping, scale transformation, and brightness and contrast perturbation, were applied during training to improve the generalization capability of the model under complex working conditions, expanding the total number of training samples to 3390. This expansion of the sample space significantly enhanced the model’s adaptability to variations in material surface morphology and imaging conditions. After the preprocessed ROI images were fed into the model, a binary segmentation mask of the deposition layer and the background could be obtained.

**Figure 4:**
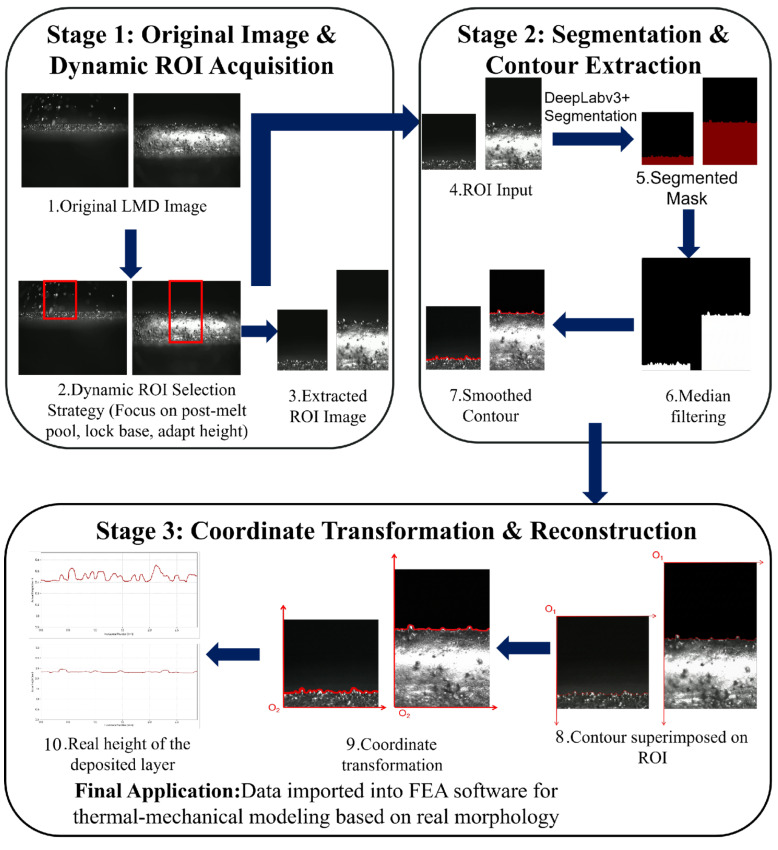
Workflow of the proposed method for deposition layer morphology extraction and full-field reconstruction, including ROI extraction, semantic segmentation, contour extraction, coordinate transformation, and profile reconstruction.

Reconstruction of the actual geometric morphology of the component requires the establishment of a mapping relationship between the image coordinate system and the physical coordinate system. Since the camera is fixed relative to the laser head, the pixel coordinates referenced to the image origin are first transformed into relative coordinates referenced to the substrate. Then, the calibrated pixel equivalent (0.0048 mm/pixel) is introduced to convert the pixel height into the actual physical height. On this basis, full-field contour stitching is performed by utilizing the spatiotemporal correlation between adjacent image frames. By calculating the horizontal pixel displacement between adjacent frames, the local contours extracted from individual frames are registered and superimposed in the horizontal direction, ultimately reconstructing the complete surface morphology of the deposition layer along the entire circular path. The reconstructed morphology was then converted into a geometric model and subsequently imported into the finite element framework to establish a thermo-mechanically coupled model. Figure 4 illustrates the proposed workflow integrating dynamic ROI locking, improved DeepLabv3+ semantic segmentation, and digital reconstruction of the deposition layer morphology.

### 2.4. Geometric Model and Finite Element Model Generation Based on Reconstructed Morphology

Based on the reconstructed morphology obtained in the previous section, a data-driven method was developed to generate the three-dimensional geometric model and the corresponding finite element model. This method utilizes the real-time melt pool position information together with the deposition layer height data extracted by deep learning algorithms, and achieves dynamic updating of the simulation geometry through the secondary development interface of finite element analysis software. Considering the solidification characteristics of the molten pool under surface tension, as well as the influence of the Gaussian distribution of laser energy and the symmetry of the coaxial powder flow, the deposition layer exhibits a macroscopic geometric feature characterized by a raised center and smooth edges. Based on the assumption that the cross-section of the deposition track follows a spherical cap shape, the local radius of curvature is inversely calculated using the measured height data, thereby constructing a three-dimensional geometric model that approximates the actual physical contour.

In the implementation phase, an initial geometric model was first established, comprising the substrate (50 mm × 50 mm × 10 mm) and the preset deposition region. The latter featured a circular track structure determined by a scanning radius of 15 mm and a track width of 2.5 mm, resulting in inner and outer diameters of 13.75 mm and 16.25 mm, respectively. Subsequently, mesh generation was performed. To fulfill the requirements of the thermo-mechanical coupled calculation, DC3D8R (8-node linear heat transfer continuum elements) and C3D8R (8-node linear brick, reduced integration) elements were assigned to the substrate and deposition layers for the thermal and mechanical analyses, respectively. The finalized numerical model consisted of 171,048 elements and 200,940 nodes.

**Figure 5:**
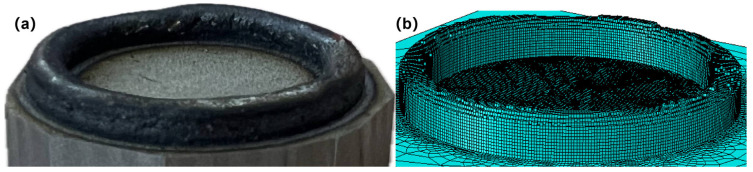
Fabricated thin-walled circular specimen and reconstructed finite element mesh model based on the actual morphology: (**a**) fabricated specimen; (**b**) reconstructed mesh model.

The execution workflow of the 3D reconstruction algorithm is as follows: First, all elements within the deposition region are traversed to extract their centroid coordinates and element indices, followed by the application of a KDTree data structure to achieve efficient spatial neighborhood retrieval. Subsequently, the algorithm progressively reads the measured height data of each sub-region to solve for the corresponding spherical center coordinates and radius parameters based on the geometric relationship of a spherical crown. Upon obtaining the key geometric parameters, the KDTree is invoked to retrieve target elements within the current deposition trajectory envelope from the set of candidate elements. To ensure the temporal accuracy of element activation, the algorithm adheres to a rigorous set operation mechanism: let S be the universal set of initial elements to be activated, and A be the union of elements activated up to the current step. In each computational increment, the algorithm only activates elements that belong to S and are located within the current geometric envelope, while simultaneously updating the remaining candidate set. To address potential empty sets caused by discretization errors (e.g., no new elements covered in a specific step), an exception handling mechanism is integrated. By outputting an empty set and maintaining the continuity of sequence indexing, the integrity and consistency of the FEA solver’s data interface are ensured [19]. The reconstructed finite element mesh model based on the actual morphology is illustrated in Figure 5. The governing equations used for the thermo-mechanical coupling simulation are provided in Appendix A.

### 2.5. Moving Heat Source Formulation and Thermo-Mechanical Coupling Strategy

In this study, a dynamic heat source loading strategy based on the actual morphology was developed to account for the non-uniform surface fluctuations of the LMD deposition layer. During the actual deposition process, the model height varies dynamically with the evolving surface morphology. To ensure the accuracy of laser energy input, the position of the double-ellipsoidal heat source must be updated in real time according to the actual height of the deposition layer.

In light of the geometric characteristics of the circular deposition path, this study defined a dynamic trajectory control equation for the heat source center. To this end, the surface height data of the deposition layer, acquired through the aforementioned visual reconstruction, were mapped onto a spatial function, $H_{recon}(x,y)$, which served as the basis for establishing the control equation. This equation characterizes the kinematic law of the heat source center under the dual constraints of the preset planar scanning path and the vision-reconstructed height field:

(1) $$P\left(t\right)=\;\{\begin{array}{c} x_{c}\left(t\right)=\;R\;\cdot cos\left(\omega t\right)\\ \;y_{c}\left(t\right)=\;R\;\cdot sin\left(\omega t\right)\\ \;z_{c}\left(t\right)=\;H_{recon}\left(x_{c}\left(t\right),\;y_{c}\left(t\right)\right)+\;\Delta h \end{array}$$

In the equations, $x_{c}\left(t\right)$ and $y_{c}\left(t\right)$ describe the circular scanning motion of the heat source on the x,y plane, where R denotes the scanning radius and ω represents the scanning velocity. $z_{c}\left(t\right)$ signifies the axial height of the heat source center, which is driven in real time by the geometry function derived from the reconstructed morphology, $H_{recon}$, established in the previous section. Additionally, Δh is the defocus amount correction coefficient (assigned as Δh = 0 in this study). This set of equations not only defines the spatial coordinates of the heat source but also constitutes the core control logic of the user subroutine employed in the numerical simulation.

In practice, the control equation is implemented via the secondary development interface of the numerical simulation software. At each time increment, the program utilizes an interpolation algorithm to retrieve the deposition layer elevation data from the environmental variables. The value of $z_{c}\left(t\right)$ in the equation is calculated and updated in real-time, thereby ensuring that the heat source center consistently and closely adheres to the actual surface of the deposition layer. This mechanism effectively eliminates the energy input deviations inherent in traditional simplified models caused by a fixed heat source height.

The thermo-mechanical coupling simulation was carried out using a sequential coupling strategy. First, transient heat transfer analysis was performed based on the dynamic heat source model described above to obtain the spatiotemporal evolution of the temperature field during the entire deposition and cooling process. Subsequently, the calculated temperature history was applied as a predefined thermal load to the mechanical model. By incorporating the temperature-dependent thermo-physical and mechanical properties of the material, the transient thermal stresses induced by non-uniform thermal expansion and contraction, as well as the final residual stress distribution, were computed.

## 3. Results and Discussion

### 3.1. Evaluation of Morphology Reconstruction Accuracy

The improved DeepLabv3+ model not only accurately identifies the overall region of the deposition layer in segmentation tasks, but also achieves refined contour extraction in boundary regions. Compared with conventional segmentation methods, the proposed model demonstrates clear advantages under complex illumination conditions, particularly in terms of boundary smoothness and detail preservation. The effectiveness of the proposed method was objectively evaluated by comparing it with PSPNet [20], YOLOv8-Seg [21], and the baseline DeepLabv3+. For fair comparison, all models were trained and evaluated using the same dataset partition, the same training epochs, and the same evaluation metrics. The evaluation metrics include mean Intersection over Union (mIoU), mean Pixel Accuracy (mPA), and overall Accuracy. The quantitative comparison results are presented in Table 2.

The improved DeepLabv3+ model achieved the best performance across all three evaluation metrics, with an mIoU of 97.32%, mPA of 98.67%, and an overall Accuracy of 99.42%. Compared with the baseline DeepLabv3+, the mIoU, mPA, and Accuracy were improved by 2.59%, 1.13%, and 0.10%, respectively. In addition, the proposed model also outperformed YOLOv8-Seg and PSPNet, demonstrating consistent superiority under the same experimental conditions. Notably, in terms of boundary accuracy, the improved model effectively mitigates the blurring and loss of edge information commonly observed in conventional methods. This improvement provides more accurate morphological input for subsequent stress modeling based on the actual deposition morphology.

**Figure 6:**
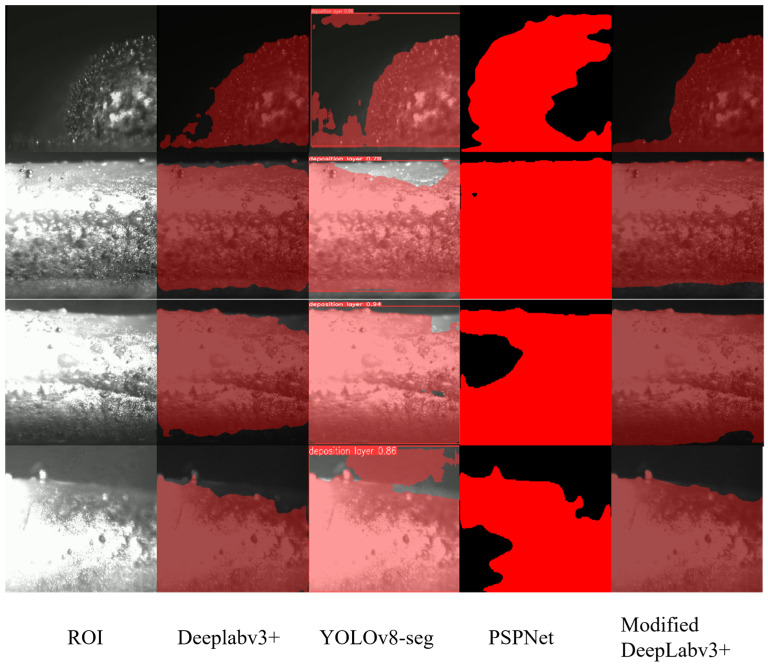
Visual comparison of segmentation results among different models under complex illumination conditions. From left to right: original image, baseline DeepLabv3+, YOLOv8-Seg, PSPNet, and the proposed improved DeepLabv3+ model.

Figure 6 presents the segmentation results obtained by different models. From left to right, the first column shows the original images of the deposition layers without segmentation. The second column displays the results of the baseline DeepLabv3+ model, which achieves relatively accurate overall boundaries but fails to capture fine details in concave regions. The third column corresponds to YOLOv8-Seg, where missed detections are observed in regions with significant illumination variations, particularly in the rear area of the deposition layer. The fourth column shows the results of PSPNet, which exhibits noticeable boundary blurring and incomplete segmentation. In contrast, the final column presents the results of the improved DeepLabv3+ model. It can be observed that the improved model demonstrates superior performance in both boundary accuracy and detail preservation. In particular, under complex illumination conditions, it is able to stably extract precise contours of the deposition layer.

**Figure 7:**
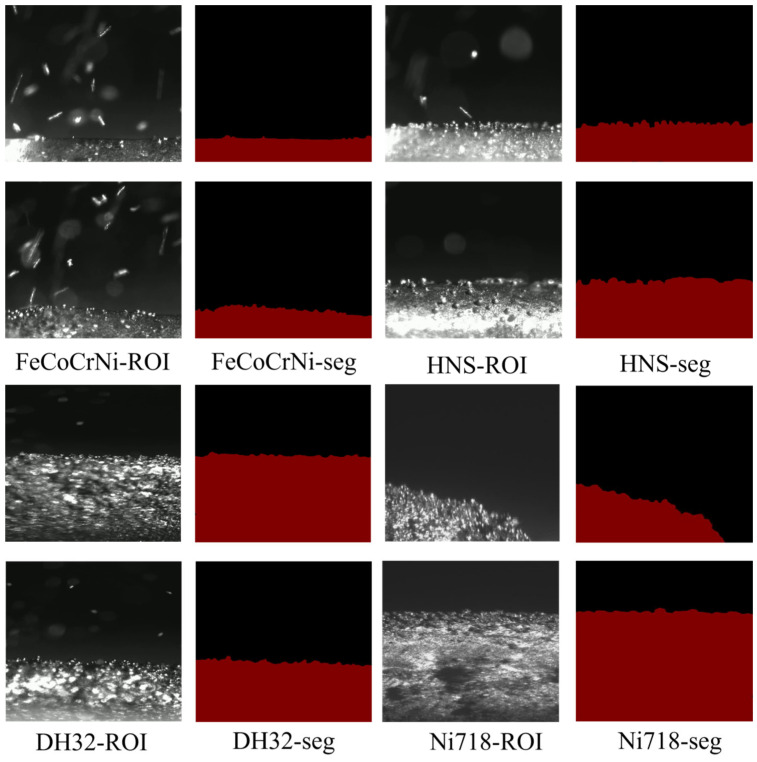
Segmentation performance on different materials with increasing deposition layers.

Figure 7 presents the segmentation performance of the improved DeepLabv3+ model across four different deposition materials, including FeCoCrNi high-entropy alloy, HNS, DH32 shipbuilding steel, and Ni718 superalloy. The experimental results demonstrate that, despite the significant differences in spatter intensity, molten pool brightness, and surface texture among these materials during the LMD process, the proposed model maintains strong robustness and stability. Specifically, for FeCoCrNi and DH32 samples with severe spatter interference, the model can accurately identify and suppress high-intensity spatter noise, effectively avoiding the generation of false edges. For HNS and Ni718 samples with strong surface reflections, the model exhibits excellent resistance to overexposure, producing binary masks with smooth boundaries that closely match the actual deposition contours. This cross-material generalization capability indicates that the model does not rely solely on material-specific visual features, but instead captures intrinsic semantic features of the deposition layer through deep residual learning. As a result, the proposed method can consistently provide high-fidelity and reliable geometric inputs for subsequent thermo-mechanical simulations, regardless of variations in material systems during continuous monitoring.

To comprehensively evaluate the accuracy and reliability of the proposed machine vision measurement system for LMD deposition layer morphology, a dial indicator and manual image analysis (based on pixel difference measurement) were employed as reference methods. The heights of ten randomly selected feature points on the deposition surface were measured and compared with the results obtained by the proposed algorithm. The detailed comparison results are presented in Table 3.

Quantitative error analysis based on the data in Table 3 indicates a high level of agreement between the measurements obtained by the proposed algorithm and the physical measurements. The errors at all monitoring points are below 0.91%, demonstrating the strong measurement stability of the method. These results confirm that the proposed approach effectively suppresses interference caused by complex conditions in the LMD process, such as stray light and powder adhesion.

**Figure 8:**
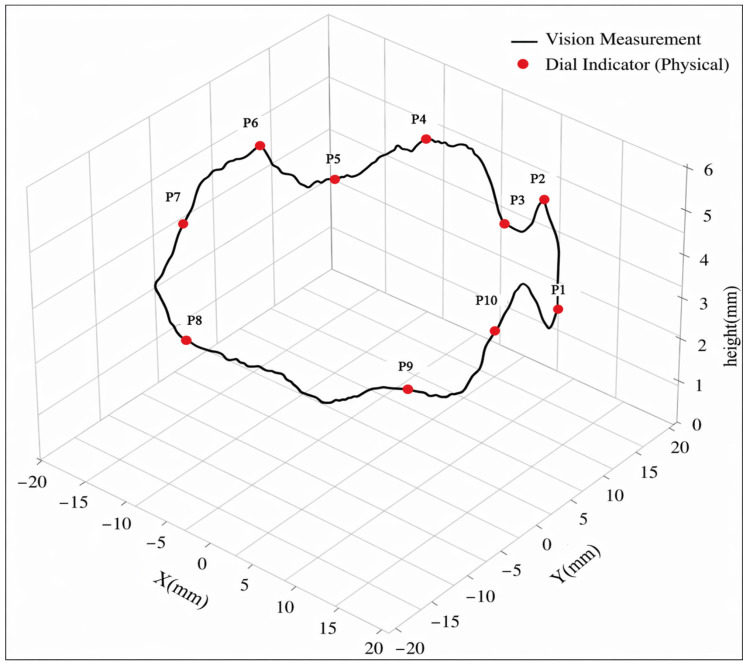
Accuracy verification and comparison of deposition layer height extraction based on machine vision.

Figure 8 further illustrates the consistency of the measurement results by presenting the reconstructed surface profile along the deposition path, where the height data obtained from three different measurement methods are mapped to the corresponding feature points. As shown in the figure, although the deposition surface exhibits pronounced peak-valley fluctuations, the proposed algorithm is able to accurately capture these fine morphological variations. The spatial distribution of the measured points shows strong agreement with both the physical measurements and the manually obtained results. The small deviations indicated by the annotation lines at each measurement point further demonstrate the accuracy of the algorithm in handling complex edge features. These results confirm the high fidelity of the proposed system in continuous morphology monitoring.

### 3.2. Validation of Thermal Field Simulation via In Situ Temperature Measurements

**Figure 9:**
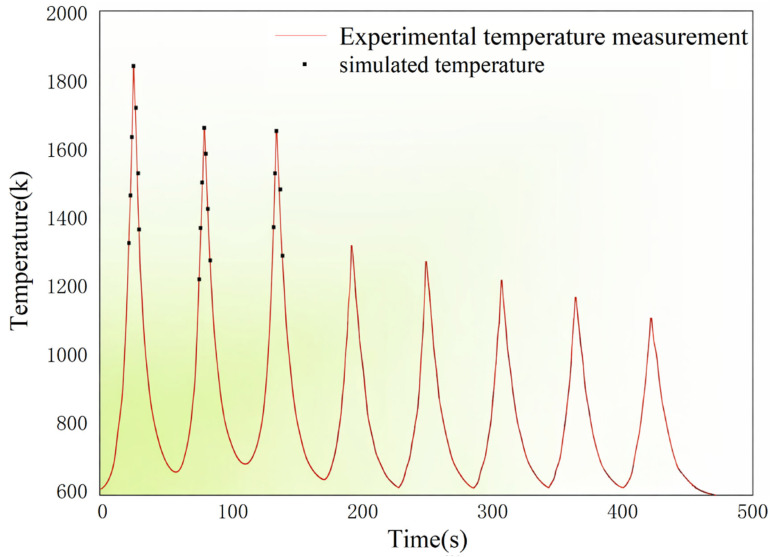
Comparison between experimentally measured and simulated thermal cycles at the monitoring point during the deposition process.

Figure 9 presents the transient temperature evolution at the monitoring point during the deposition process, with a full-cycle comparison between the numerical simulation results and the infrared temperature measurement data. The results show that the simulated temperature curve successfully captures the characteristic periodic thermal cycles of the LMD process. In addition, the peak temperature points predicted by the simulation are in close temporal agreement with the experimental measurements. From the overall evolution trend, the simulation results not only match the experimental data in terms of waveform characteristics but also exhibit a high degree of consistency in the cooling stage, particularly in the cooling rate reflected by the slope of the temperature curve.

### 3.3. Evolution of Residual Stress and Morphology-Induced Mechanisms

**Figure 10:**
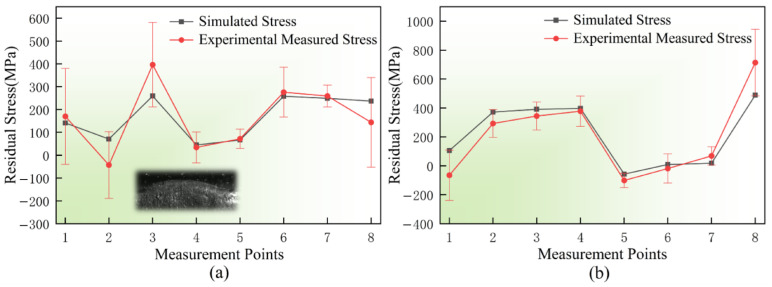
Comparison of simulated and experimental tangential residual stress distributions under different laser powers (The inset highlights the local morphology at Point 3): (**a**) 1000 W; (**b**) 1500 W.

Figure 10 presents the comparison of tangential residual stresses at different measurement points under two laser power conditions, 1000 W (Figure 10a) and 1500 W (Figure 10b). Under both thermal input conditions, the stress values predicted by the numerical simulation show good agreement with the XRD experimental measurements in terms of both magnitude and variation trend. This result provides strong validation for the thermo-mechanical coupling model established based on the actual deposition morphology, demonstrating its effectiveness and reliability in predicting residual stress distributions in the LMD process.

For the stress distribution under a laser power of 1000 W (Figure 10a), it can be observed that the stress value at the third measurement point is significantly higher than that at the adjacent regions. As indicated by the locally magnified morphology embedded in Figure 10a, this point is located at a pronounced surface protrusion of the deposition layer. Such abrupt geometric variation induced by the actual surface morphology leads to significant local stress concentration, which is identified as the primary factor responsible for the stress peak at this location.

In contrast, under a higher laser power of 1500 W (Figure 10b), the stress evolution exhibits higher peak values and more pronounced fluctuations. On the one hand, the increased heat input enlarges the molten pool size and raises the peak temperature, causing more severe overall contraction during the cooling stage, which leads to higher residual stress levels. On the other hand, the elevated thermal input significantly amplifies the influence of micro-scale surface morphology on heat dissipation and stress distribution. This enhanced coupling effect results in greater fluctuations in the stress curve compared with the 1000 W condition [22].

**Figure 11:**
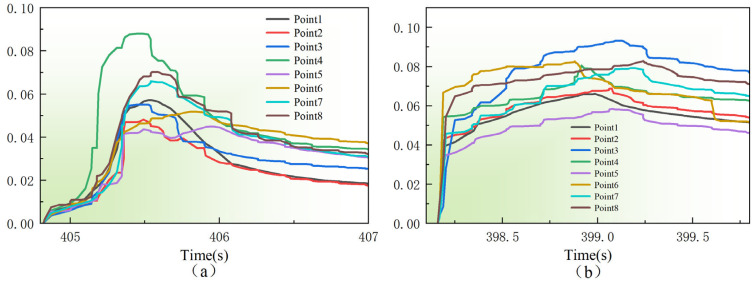
Transient thermal strain evolution history at monitoring points: (**a**) 1000 W; (**b**) 1500 W.

Figure 11 presents the transient strain evolution at representative locations of the deposition layer under laser powers of 1000 W (Figure 11a) and 1500 W (Figure 11b) to further clarify the physical origin of the differences in stress distribution. It should be noted that measurement points 1–8 in Figure 11 correspond exactly to the sampling locations used for XRD residual stress analysis in Figure 10. This correspondence is intended to reveal the formation mechanism of residual stress through a consistent analysis of the strain evolution history.

Under the laser power of 1000 W (Figure 11a), the strain evolution curves at different measurement points exhibit good overall consistency, showing a typical pattern of rapid increase, sharp decrease, and gradual stabilization. This indicates that, under relatively low heat input, the deposition layer undergoes intense transient thermal expansion followed by rapid cooling contraction, resulting in a short thermal cycle and a relatively uniform heat-affected zone. Notably, measurement point 3 exhibits a pronounced strain peak during the transient evolution. As revealed by the morphological features in Figure 10a, this point corresponds to a distinct surface protrusion of the deposition layer. Such geometric irregularity not only alters the local heat dissipation conditions but also induces more severe constrained contraction during the thermal cycle, leading to significant plastic strain accumulation. This dynamic evolution process provides a physical explanation for the stress concentration observed at point 3 in Figure 10a, demonstrating a strong positive correlation between transient strain peaks and residual stress maxima [23,24].

In contrast, under a higher laser power of 1500 W (Figure 11b), the strain evolution curves show significantly increased divergence, with most measurement points exhibiting a slower decrease after reaching their peak values. The underlying mechanism can be attributed to two main factors. First, the higher energy input at 1500 W leads to a substantial increase in the molten pool temperature and the extent of the heat-affected zone, resulting in intensified thermal accumulation within the material. This excess heat prolongs the residence time of the material in the high-temperature plastic regime, giving rise to a pronounced high-strain plateau. Second, the elevated heat input induces steeper temperature gradients within the deposition layer, causing asynchronous thermodynamic responses at different locations during the cooling and contraction process. This non-uniform plastic deformation, triggered by high power input, leads to divergent strain evolution paths among the measurement points, in contrast to the more consistent behavior observed under the 1000 W condition.

**Figure 12:**
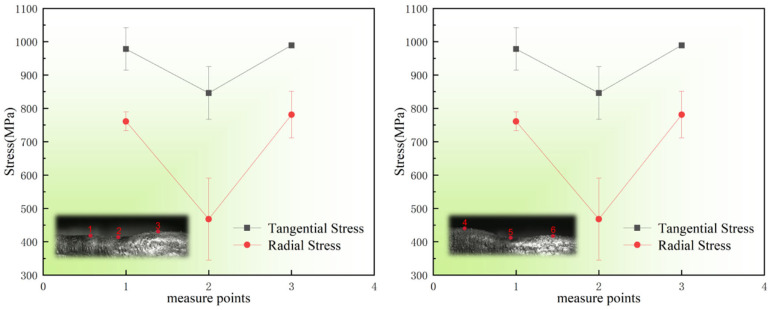
Comparison of tangential and radial residual stresses at typical morphological feature points (The insets show the corresponding convex and concave positions): Points 2 and 5 are concave regions; Points 1, 3, 4, and 6 are convex regions.

To further investigate the specific influence of local morphology on stress distribution, an additional sample with more pronounced surface features was selected for analysis. Two typical concave regions and four adjacent convex regions on the deposition surface were identified as characteristic locations. The corresponding residual stress distribution at these points is presented in Figure 12.

Experimental observations indicate that the residual stress level in convex regions is significantly higher than that in concave regions. The peak stress in convex areas reaches up to 989 MPa, whereas the maximum value in concave regions is only 778 MPa. The underlying mechanism for this difference can be attributed to two main factors. First, the concave regions are located between adjacent high-temperature convex areas, where the geometric configuration restricts heat dissipation pathways, resulting in relatively delayed cooling. In contrast, convex regions, benefiting from more favorable heat dissipation conditions, enter the rapid cooling stage earlier. The higher cooling rate intensifies local temperature gradients, causing convex regions to undergo thermal contraction sooner. This asynchronous cooling behavior leads to significant incompatibility in shrinkage between adjacent regions, where the free contraction of convex areas is constrained by the surrounding high-temperature material, thereby generating substantial tensile stress [25]. Second, considering the relatively high coefficient of thermal expansion of HNS, the greater local height of convex regions results in larger volumetric shrinkage during cooling. Since the deposition layer behaves as a continuous body, this spatial mismatch in shrinkage induced by geometric height differences leads to additional mechanical constraint. The regions with greater shrinkage (convex areas) are subjected to geometric restraint from neighboring regions with smaller shrinkage (lower-height areas), thereby inducing more severe tensile stress concentration [26]. In contrast, concave regions, characterized by limited heat dissipation area, slower cooling rates, and compressive constraints from surrounding material, exhibit relatively lower residual stress levels [27].

The significant stress differences observed among regions with different morphologies further highlight the critical importance of acquiring high-fidelity surface contours during LMD process monitoring, which also serves as the primary motivation for the improved vision algorithm proposed in this study. Even minor deviations in surface morphology may be amplified in thermo-mechanical coupling simulations, leading to inaccurate prediction of stress peaks. The improved DeepLabv3+ network developed in this work is capable of extracting clear, continuous, and high-precision deposition layer contours even under strong illumination and spatter interference. This enables the finite element model to incorporate accurate geometric inputs, thereby capturing stress variations induced by subtle morphological changes. Therefore, high-precision visual reconstruction of surface morphology is not only the foundation of three-dimensional reconstruction, but also a prerequisite for ensuring the reliability of residual stress prediction.

In addition, Figure 12 compares the tangential and radial residual stresses at different measurement points. Due to the unique ring-shaped geometry, the thermal expansion and contraction behavior of the sample is subject to significant geometric constraints. While deformation in the radial and axial directions is relatively unconstrained, the tangential deformation is strongly restricted by the closed-loop nature of the circular structure. This geometric constraint leads to the accumulation of stress predominantly in the tangential direction, resulting in tangential stresses that are significantly higher than those in the radial and axial directions [28].

**Figure 13:**
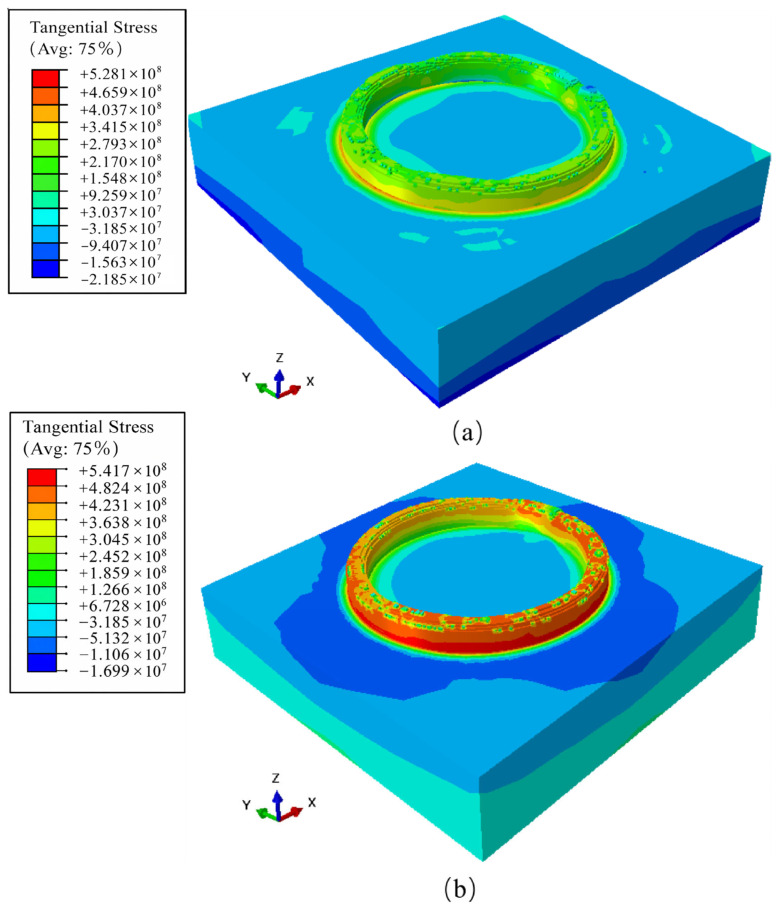
Macroscopic 3D distribution of tangential residual stress under different laser powers: (**a**) 1000 W; (**b**) 1500 W.

Based on the understanding of the local morphology-induced mechanisms, the macroscopic distribution and local concentration behavior of residual stress in the deposited component were further analyzed using the stress contour maps (Figure 13a,b). The results indicate a pronounced gradient of residual stress along the deposition direction, with more significant stress concentration occurring in the bottom region. This phenomenon can be attributed to the strong constraint imposed by the substrate. The bottom layers are firmly bonded to the substrate and therefore experience restricted shrinkage during cooling, which leads to the accumulation of higher residual stresses. In contrast, the material in the upper region is less constrained by the substrate and undergoes relatively slower cooling, allowing stress to be more effectively relaxed during the cooling process [29,30].

Under the higher laser power of 1500 W (Figure 13b), the stress distribution exhibits greater spatial heterogeneity compared with the 1000 W condition (Figure 13a). This behavior is primarily attributed to the elevated local temperatures induced by increased laser power. The higher heat input amplifies the influence of surface morphology on heat dissipation pathways. In thicker deposition regions, heat dissipation is delayed, whereas thinner regions cool more rapidly. This asynchronous cooling and shrinkage behavior introduces strong mechanical interactions between adjacent regions, leading to intensified constraint effects. Furthermore, due to the relatively high yield strength of HNS, the local plastic mismatch induced by thermal lag cannot be effectively relieved through deformation accommodation. As a result, these combined effects lead to a more pronounced spatial dispersion of residual stress under high-power conditions [31].

## 4. Conclusions

1. An improved DeepLabv3+ semantic segmentation model was proposed to address the extreme interference conditions in LMD processes, including strong light reflection, intense molten pool radiation, and spatter noise. By employing ResNet101 as the backbone network instead of a lightweight architecture, the model effectively alleviates gradient vanishing issues and enhances the extraction of fine-grained features. In addition, the integration of the DenseASPP module and a cross-level feature fusion mechanism enables an effective combination of low-level spatial details and high-level semantic information. These improvements significantly enhance the robustness of the model under low-contrast and complex background conditions, achieving pixel-level accurate extraction of deposition layer contours.
2. The proposed model achieves the best performance across all evaluation metrics, with a mean Intersection over Union (mIoU) of 97.32%, a mean Pixel Accuracy (mPA) of 98.67%, and an overall Accuracy of 99.42%. Compared with the baseline DeepLabv3+ model, the mIoU is improved by 2.59%. In addition, the proposed model outperforms state-of-the-art methods such as YOLOv8-Seg and PSPNet in both boundary detail representation and overall shape recognition. These results demonstrate its superior performance and strong potential for in situ monitoring in additive manufacturing.
3. The machine vision-based method for extracting deposition layer height demonstrates high accuracy, with all measurement errors controlled within 0.91% when compared with dial indicator measurements. This high-fidelity morphological data provides reliable boundary conditions for subsequent thermo-mechanical simulations, overcoming the limitations of traditional idealized models that neglect micro-scale morphological features, and laying a solid foundation for accurate prediction of residual stress evolution.
4. A comparison between the XRD-measured residual stresses and the numerical simulation results reveals a high degree of consistency in both magnitude and distribution trends, thereby validating the accuracy of the proposed “monitoring-reconstruction-simulation” coupled methodology. Notably, in regions with significant topographical fluctuations, the model based on actual morphology accurately captures the localized stress peaks induced by morphological variations, which remain unpredictable by traditional simplified models based on idealized flat-layer assumptions. These findings demonstrate the necessity of incorporating real-time morphological data into simulations, particularly for stress-sensitive materials such as HNS.
5. This study reveals the significant influence of local morphology on residual stress distribution in HNS laser additive manufacturing. Convex regions, characterized by larger heat dissipation areas and higher cooling rates, undergo rapid shrinkage that is strongly constrained by the surrounding material, leading to pronounced stress concentration. In contrast, concave regions exhibit slower heat dissipation and more gradual cooling, which reduces local temperature gradients and mitigates thermal stress accumulation, resulting in lower residual stress levels. Furthermore, due to the geometric constraint imposed by the closed-loop ring structure, tangential stress is significantly higher than radial stress.

## Figures and Tables

**Table 1:** Chemical composition of the HNS powder (wt%) [14].

Components	C	N	Cr	Ni	Mn	Mo	Cu	O	Fe
Amount	0.035	0.42	18.96	0.16	12.6	2.97	0.35	0.07	Balance

**Table 2:** Quantitative comparison of segmentation performance among different models.

Model	mIoU (%)	mPA (%)	Accuracy (%)
Modified DeepLabv3+	97.32	98.67	99.42
DeepLabv3+	94.73	97.64	99.32
YOLOv8-Seg	94.71	97.71	96.71
PSPNet	95.69	97.30	98.40

**Table 3:** Comparison of deposition layer height measurements obtained by different methods.

Measurement Point	Developed Algorithm (mm)	Dial Indicator (mm)	Manual Image Analysis (mm)
1	3.605 ± 0.006	3.638	3.618 ± 0.048
2	4.770 ± 0.004	4.764	4.762 ± 0.058
3	3.549 ± 0.005	3.542	3.552 ± 0.033
4	4.860 ± 0.007	4.854	4.848 ± 0.043
5	3.735 ± 0.008	3.766	3.740 ± 0.029
6	4.806 ± 0.003	4.849	4.818 ± 0.067
7	3.980 ± 0.006	3.968	3.982 ± 0.038
8	3.702 ± 0.005	3.731	3.714 ± 0.052
9	3.781 ± 0.004	3.772	3.786 ± 0.024
10	4.508 ± 0.007	4.545	4.520 ± 0.062

**Figure A1:**
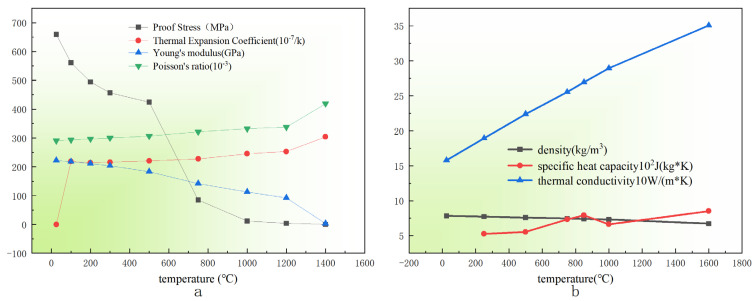
Temperature-dependent material properties of HNS used in the simulation: (**a**) Mechanical properties; (**b**) Thermophysical properties.

## Data Availability

The original contributions presented in this study are included in the article. Further inquiries can be directed to the corresponding author.

## Appendix A. Governing Equations for Thermo-Mechanical Coupling Simulation

During the LMD process, the material undergoes intense rapid heating and non-equilibrium solidification, where the evolution of the internal temperature field directly dictates the formation of thermal stresses. To accurately describe this process, a three-dimensional governing equation incorporating a moving heat source term was established based on transient heat conduction theory. According to the law of conservation of energy, the governing equation for transient heat conduction in an isotropic material can be expressed as [32]:

(A1) $$\rho C_{p}\frac{\partial T}{\partial t}=\nabla \cdot \left(k\nabla T\right)+Q$$

where ρ is the material density (${\mathrm{kg}/m}^{3}$), $C_{p}$ is the specific heat capacity (J/(kg⋅K)), T is the temperature (K), t is the time(s), k is the thermal conductivity (W/(m⋅K)) and Q is the internal heat source generation rate (${W/m}^{3}$). The left side of the equation represents the rate of change in internal energy per unit volume of the material over time. On the right side, the first term denotes the divergence of heat flux caused by thermal conduction, while the second term, Q, represents the laser energy input.

To accurately simulate the heating effect of the moving laser beam on the substrate and the deposition layers, the Goldak double-ellipsoid heat source model was adopted in this study. This model accounts for the discrepancy in temperature gradients between the front and rear ends of the molten pool by describing the heat flux distribution as two semi-ellipsoids. The heat source distribution in the front semi-ellipsoid, denoted as $Q_{f}\left(x,y,z\right)$ is expressed as [33]:

(A2) $$Q_{f}\left(x,y,z\right)=\frac{6\sqrt{3}f_{f}P\eta }{abc_{f}\pi \sqrt{\pi }}\exp\left(-\frac{3x^{2}}{a^{2}}-\frac{3y^{2}}{b^{2}}-\frac{3z^{2}}{c_{f}^{2}}\right)$$

Similarly, the heat source distribution in the rear semi-ellipsoid, denoted as $Q_{r}\left(x,y,z\right)$, is expressed as:

(A3) $$Q_{r}\left(x,y,z\right)=\frac{6\sqrt{3}f_{r}P\eta }{abc_{r}\pi \sqrt{\pi }}\exp\left(-\frac{3x^{2}}{a^{2}}-\frac{3y^{2}}{b^{2}}-\frac{3z^{2}}{c_{r}^{2}}\right)$$

where x, y, z are the coordinates in the local coordinate system with the center of the heat source as the origin; P is the laser power (W), and η is the laser absorption rate. $f_{f}$ and $f_{r}$ denote the energy distribution coefficients for the front and rear semi-ellipsoids, respectively, satisfying $f_{f}+f_{r}=2$. The parameters $a,b,c_{f},c_{r}$ represent the shape parameters of the molten pool in terms of width, depth, and front/rear length, respectively; these parameters are calibrated based on the actual morphological characteristics of the molten pool.

During the solution process, at the initial time t=0, the entire computational domain is assumed to be at a uniform ambient temperature $T_{0}=298\;K$. Throughout the deposition process, convective heat transfer and thermal radiation occur simultaneously between the workpiece surface and the surrounding environment. Therefore, a combined thermal boundary condition is employed to describe the energy loss from the workpiece surface [34]:

(A4) $$-k\frac{\partial T}{\partial n}=h_{c}\left(T_{s}-T_{\infty }\right)+\varepsilon \sigma \left(T_{s}^{4}-T_{\infty }^{4}\right)$$

where n is the surface normal vector, $h_{c}$ is the convective heat transfer coefficient $W/\left(m^{2}\cdot K\right)$, $T_{s}$ is the workpiece surface temperature, $T_{\infty }$ is the ambient temperature,ε is the surface emissivity, and σ is the Stefan-Boltzmann constant ($5.67\times 10^{-8}\;W/\left(m^{2}\cdot K^{4}\right)$).
